# Multi-Modal Profiling Reveals SERPINB3-Driven Immune Evasion and Stromal Immune Mimicry in Triple-Negative Breast Cancer

**DOI:** 10.3390/genes17010038

**Published:** 2025-12-31

**Authors:** Zinab O. Doha

**Affiliations:** 1 Department of Clinical Laboratory Sciences, College of Applied Medical Sciences, Taibah University, Madinah 42353, Saudi Arabia; ztoha@taibahu.edu.sa; Tel.: +966-548542170; 2 Health and Life Research Center, Taibah University, Madinah 42353, Saudi Arabia

**Keywords:** triple-negative breast cancer, tumor microenvironment, single-cell RNA sequencing, immune evasion, SER-PINB3, immune mimicry, multiplex tissue imaging

## Abstract

Background/Objectives: Triple-negative breast cancer (TNBC) exhibits high immune infiltration yet remains clinically aggressive. Although immune checkpoint blockade benefits a subset of patients, the molecular programs enabling concurrent immune activation and immune evasion in TNBC are not fully defined. This study aimed to identify TNBC-specific tumor-intrinsic and tumor-extrinsic molecular features that may explain this paradox. Methods: Publicly available single-cell RNA-sequencing data from primary breast tumors were analyzed to characterize subtype-specific transcriptional programs across epithelial and stromal compartments. Tumor-intrinsic findings were independently validated using bulk transcriptomic and clinical data from the METABRIC cohort. Tumor microenvironment remodeling was evaluated using multiplexed tissue imaging of TNBC tumors. Functional analyses were done included Gene Ontology enrichment, Hallmark gene set enrichment analysis, and SERPINB3-centered protein–protein interaction network analysis using STRING. Results: Single-cell analysis identified *SERPINB3* as a TNBC-enriched epithelial gene relative to ER+ and HER2+ tumors. This subtype-restricted pattern was validated in the METABRIC cohort and associated with pathways related to epithelial–mesenchymal transition, interferon signaling, and antigen presentation. TNBC tumors also displayed a humoral immune signature characterized by B-cell and plasmablast enrichment, as well as ectopic immunoglobulin gene expression in cancer-associated fibroblasts, endothelial cells, and myeloid populations. Multiplex imaging revealed coordinated associations between immune suppression, stromal activation, and tumor proliferation. Network analysis placed SERPINB3 within interconnected immune-regulatory and stromal signaling modules. Conclusions: Together, these data indicate that TNBC exhibits co-existing immune activation and immune-suppressive features. The identified epithelial and stromal signatures represent candidate biomarkers that may inform future studies of immune regulation and therapeutic stratification in TNBC.

## 1. Introduction

Triple-negative breast cancer (TNBC) accounts for approximately 10–20% of all breast cancers and is defined by the absence of estrogen receptor (ER), progesterone receptor (PR), and HER2 expression. This molecular profile not only serves as a diagnostic hallmark but also renders TNBC unresponsive to endocrine therapies and HER2-targeted treatments, leaving chemotherapy as the primary standard of care [1,2]. Although several innovative therapeutic strategies have recently emerged for TNBC—including antibody–drug conjugates, and PARP inhibitors—these approaches benefit only a subset of patients, and therapeutic resistance frequently develops. As a result, TNBC is often associated with early relapse, visceral metastasis, and poor long-term survival. These challenges indicate the urgent need to better understand the distinct biology of TNBC and identify actionable molecular targets that could improve patient outcomes in this aggressive subtype [3].

Additionally, immune checkpoint blockade has transformed cancer therapy; however, its clinical benefit in triple-negative breast cancer (TNBC) remains inconsistent and limited to a subset of patients [4]. Despite TNBC being one of the most immunogenic breast cancer subtypes, with frequent immune infiltration and elevated immune signaling, response rates to PD-1/PD-L1 inhibitors are modest and durable benefit is observed in only a fraction of cases [5]. This variability reflects pronounced immune heterogeneity within TNBC, characterized by diverse immune cell compositions, activation states, and spatial organization across tumors [6,7]. These limitations reflect both intrinsic and microenvironmental resistance mechanisms, indicating that immune checkpoint expression alone does not fully capture the regulatory programs governing immune activation, immune suppression, and tumor progression in TNBC [6]. TNBC employs multiple immune evasion mechanisms, including aberrant immune checkpoint expression, metabolic reprogramming, epigenetic regulation, and active remodeling of the tumor microenvironment through immunosuppressive stromal and myeloid populations, collectively enabling escape from effective antitumor immunity [7]. Consequently, identifying additional immune-regulatory mechanisms and context-specific molecular targets remains essential for improving patient stratification and designing more effective combinatorial immunotherapies.

While recent immune-profiling studies have cataloged immune cell populations and activation states in TNBC, many remain largely descriptive and do not resolve how tumor-intrinsic transcriptional programs actively shape immune behavior and stromal remodeling. In particular, it remains unclear how TNBC can simultaneously exhibit robust immune activation while sustaining immune evasion and aggressive growth. Addressing this paradox requires integrative frameworks that move beyond immune cell abundance toward identifying tumor-intrinsic drivers and stromal signaling networks that modulate immune function.

Emerging frameworks for immune-target discovery emphasize that durable responses to cancer immunotherapy require identifying tumor-intrinsic signaling programs and stromal pathways that regulate immune surveillance, immune escape, and therapeutic resistance, beyond canonical checkpoint molecules alone. Recent work highlights the central roles of immune-regulatory signaling networks—including JAK/STAT, interferon, MAPK, and PI3K–AKT–mTOR pathways—as well as stromal components such as cancer-associated fibroblasts (CAFs) and endothelial cells, in shaping immune exclusion and immunotherapy failure [8]. These insights underscore the need for integrative approaches that connect epithelial transcriptional states with immune and stromal signaling logic, rather than relying solely on immune cell phenotyping or checkpoint expression.

Consistent with these emerging paradigms, this study aims to identify TNBC-specific molecular programs that couple tumor-intrinsic transcriptional reprogramming to immune modulation and stromal remodeling. By integrating single-cell transcriptomics with independent clinical and spatial validation, we position our analysis within contemporary immune-target discovery frameworks to uncover context-specific immune-regulatory mechanisms relevant for biomarker development and rational combination immunotherapy, thereby advancing immune-target discovery beyond descriptive immune profiling.

Here, we integrated secondary single-cell RNA-sequencing data from primary breast tumors [9] with clinical validation from the METABRIC cohort [10] and tissue-level multiplex imaging [11] to define TNBC-specific molecular programs. Using a bi-volcano framework to isolate genes consistently enriched in TNBC relative to ER+ and HER2+ tumors, we identified *SERPINB3* as a uniquely upregulated cancer epithelial marker, which we validated in clinical METABRIC tumors. TNBC also demonstrated a distinct humoral signature, including plasmablast activation and ectopic immunoglobulin expression in CAFs, endothelial, and myeloid cells—an immune mimicry phenotype mediating immune suppression not observed in other subtypes. Imaging data confirmed that high immune suppression marks strongly correlate with stromal activation and proliferation, reflecting tumor-extrinsic consequences of this reprogramming. STRING network analysis further positioned SERPINB3 as a mechanistic hub linking tumor-intrinsic signaling to stromal remodeling and immune evasion. Collectively, these integrative analyses reveal that TNBC simultaneously drives immune activation and immune suppression, providing a mechanistic explanation for its paradoxical behavior as both highly immunogenic and clinically aggressive, and highlighting new targets for precision immunotherapy.

## 2. Materials and Methods

### 2.1. Data Sources and Ethics

This study integrates complementary, publicly available datasets to interrogate TNBC biology across two levels: tumor-intrinsic transcriptional programs (cancer epithelial states) and tumor-extrinsic microenvironmental remodeling (immune–stromal architecture). Discovery analyses were performed using a single-cell RNA-sequencing (scRNA-seq) Breast Cancer Atlas dataset (GEO: GSE176078) [9], comprising 26 primary breast tumors (TNBC *n* = 9, ER+ *n* = 13, HER2+ *n* = 4) with curated cell-type annotations, enabling cell-type–resolved identification of TNBC-enriched epithelial and stromal signatures. To test whether key tumor-intrinsic signals identified at single-cell resolution generalize to independent patient cohorts, we validated subtype-associated expression patterns using the METABRIC dataset (*n* = 2509 primary breast cancers; TNBC *n* = 335, ER+ *n* = 1825, HER2+ *n* = 247) [10]. To validate whether the predicted microenvironmental programs correspond to measurable tissue-level states, we further analyzed a multiplexed imaging cohort of TNBC tumors (*n* = 38; 49 markers) providing spatially resolved quantification of immune suppression, stromal activation, and proliferation markers alongside clinical outcomes [11].

All data were obtained from publicly accessible repositories and were fully de-identified. No new patient recruitment or intervention was performed; therefore, additional institutional ethics approval was not required.

### 2.2. scRNA-Seq Preprocessing and Cell-Type Stratification

Single-cell RNA-sequencing data were analyzed using Seurat v5 [12] in R (v4.3.1). To minimize technical variability and avoid reintroducing batch-dependent artifacts, cell clustering and lineage annotations were adopted directly from the original study [9], which applied standardized preprocessing, quality control, and canonical marker-based cell-type assignment. This approach ensured consistency with prior biological validation and avoided overfitting cell identities to downstream subtype comparisons.

For downstream analyses, cells were stratified by major annotated lineages, including cancer epithelial cells, T cells, B cells, plasmablasts, myeloid cells, cancer-associated fibroblasts (CAFs), and endothelial cells. Differential expression analyses were performed within each lineage independently, rather than across pooled cell populations, to control for cell-type composition differences across tumor subtypes and to isolate subtype-specific transcriptional programs within comparable cellular contexts. This lineage-resolved strategy reduces confounding from cell-type imbalance and enables direct comparison of tumor-intrinsic versus tumor-extrinsic programs.

### 2.3. Differential Expression and Bi-Volcano Visualization

Differential gene expression was assessed within each cell type using Seurat’s FindMarkers function with the Wilcoxon rank-sum test, comparing TNBC cells separately against HER2+ and ER+ cells. Importantly, HER2+ and ER+ tumors were not aggregated into a single “non-TNBC” comparator, as these subtypes are driven by distinct oncogenic signaling programs (growth factor receptor–driven versus hormone receptor–driven). Aggregation would therefore risk masking subtype-specific contrasts and inflating false negatives.

To address this, we implemented a bi-volcano visualization framework that integrates two independent differential expression comparisons—TNBC vs. HER2+ and TNBC vs. ER+—into a single two-dimensional representation. For each gene, the average log_2_ fold change from TNBC versus ER+ was plotted on the x-axis, and the corresponding value from TNBC versus HER2+ was plotted on the y-axis. This design enables direct visualization of genes that are consistently altered in TNBC relative to both receptor-positive subtypes, while preserving the biological independence of each comparison.

This framework offers several advantages over conventional single-contrast volcano plots. First, it reduces reliance on arbitrary grouping of heterogeneous subtypes. Second, it provides intrinsic robustness to noise, as genes must show concordant directionality across two independent comparisons to be classified as TNBC-enriched or depleted. Third, it facilitates intuitive discrimination between subtype-restricted, partially shared, and nonspecific transcriptional changes without imposing additional statistical thresholds beyond those used in the underlying differential expression tests.

Genes located in the upper-right quadrant (positive log_2_ fold change in both comparisons) were classified as TNBC-enriched, whereas genes in the lower-left quadrant were considered consistently downregulated in TNBC. Genes aligned with a single axis reflected subtype-specific differences relative to only one comparator, and genes near the origin showed minimal differential expression. Genes were prioritized for downstream analyses based on concordant directionality and statistical significance across both comparisons. Visualization was performed using ggplot2, dplyr, and ggrepel in R software, version 4.2.3.

### 2.4. Functional Enrichment and Gene Set Analyses

To evaluate the functional relevance of differentially expressed genes, we conducted Gene Ontology Biological Process enrichment analysis using the clusterProfiler R package (v4.0.5) [13]. Enrichment analyses were performed separately for upregulated and downregulated gene sets, allowing directional interpretation of TNBC-associated programs rather than collapsing opposing biological effects. Enrichment analysis was performed using the enrichGO() function with Benjamini–Hochberg false discovery rate (FDR) correction to control for multiple testing. Visualization of enrichment results was carried out using the dotplot(), cnetplot(), and heatplot() functions from the enrichplot package (v1.3.4).

To assess pathway-level activity and hallmark signaling programs, we conducted GSEA using the clusterProfiler package (v4.0.5) in R. Pre-ranked gene lists were generated based on log2 fold change values alone from TNBC versus HER2+ and TNBC versus ER+ comparisons within the cancer epithelial cell compartment. Enrichment analysis was performed against the MSigDB Hallmark gene sets [14], which provide compact, non-redundant representations of core oncogenic and immune signaling pathways. This approach avoids arbitrary gene cutoffs and enables detection of subtle but coordinated transcriptional shifts. Results were visualized using the ridgeplot() function from the enrichplot package. Pathways with an adjusted *p* value (FDR) < 0.05 were considered significantly enriched.

### 2.5. Pseudo-Bulk Expression Profiling

To evaluate lineage-specific expression patterns of immunoglobulin genes while minimizing single-cell sparsity and dropout effects, we generated pseudo-bulk expression profiles by aggregating single-cell transcriptomes within each annotated cell type. Average expression values were computed per cell type using the AggregateExpression() function in Seurat v5, following log-normalization of the dataset with NormalizeData(). Immunoglobulin variable region genes (IGLV3-25, IGLV1-40, IGKV1-9, IGLV7-46) were selected based on their prominence in differential expression analyses. The DotPlot() function was then used to simultaneously display the relative expression level (color intensity) and the proportion of expressing cells (dot size) for each gene across annotated cell types, including plasmablasts, B cells, cancer epithelial cells, CAFs, myeloid, endothelial, and other relevant populations.

### 2.6. METABRIC Clinical Cohort Analysis

To independently validate tumor-intrinsic transcriptional findings at the patient level, we analyzed bulk transcriptomic and clinical data from the METABRIC cohort (*n* = 2509 primary breast cancers) [10]. *SERPINB3* expression values were accessed through cBioPortal for all samples and stratified by molecular subtype (TNBC, ER+, HER2+). Expression frequency rather than absolute expression level was used as the primary metric to assess subtype enrichment, reflecting the known heterogeneity of TNBC.

To evaluate tumor-intrinsic pathway associations, *SERPINB3*-altered TNBC cases were analyzed using the cBioPortal “Enrichment” and “Pathway Analysis” tools. Pathways were ranked by adjusted *p*-value, and the top enriched functional categories were visualized and compared with those identified in the single-cell analyses. This strategy allowed assessment of concordance between single-cell–derived epithelial programs and bulk tumor biology, while acknowledging that bulk data capture averaged signals across mixed cell populations.

### 2.7. Multiplex Imaging Validation

To test whether transcriptionally inferred immune and stromal programs correspond to measurable tissue-level states, we analyzed a multiplexed imaging dataset of TNBC tumors (*n* = 38; 49 markers) [11]. Rather than focusing on individual markers, we constructed biologically informed composite indices representing immune suppression, CAF activation, endothelial activity, and proliferation. This index-based approach reduces marker-specific noise and captures coordinated functional programs relevant to tumor–immune interactions. From this dataset, we derived biologically informed composite indices representing key functional programs: immune suppression (PD-L1, PD-1, LAG3, FOXP3, IDO, CD68), CAF activity (SMA, Vimentin), endothelial activity (CD31), and proliferation (Ki67). Markers were not thresholded at the individual-marker level. For each patient, marker expression values were standardized by z-scoring and averaged within each marker set to generate composite indices. Associations between indices were evaluated using Spearman correlation coefficients, with results visualized through scatterplots and correlation heatmaps generated in the ggpubr and corrplot R packages. To assess clinical relevance, patients were stratified into “high” versus “low” groups for the immune suppression index using the median value as cutoff. Kaplan–Meier analyses of overall survival (OS) and recurrence-free survival (RFS) were then performed, incorporating log-rank tests and confidence intervals, using the survival and survminer packages.

### 2.8. STRING Protein–Protein Interaction Analysis

To investigate whether *SERPINB3* could mechanistically link tumor-intrinsic transcriptional programs to stromal activation and immune suppression, the TNBC-exclusive epithelial gene set identified from the scRNA-seq analysis (Supplementary Dataset Table S3) was uploaded into the STRING protein–protein interaction database (v12.0). Protein–protein interactions (PPIs) were filtered to retain only SERPINB3-associated interactions and high-confidence PPIs, defined by a STRING combined confidence score > 0.7. A SERPINB3-centered interaction network was generated, and protein clusters were annotated based on biological function.

Gene Ontology (GO) Biological Process enrichment was performed on the SERPINB-linked cluster using STRING’s integrated enrichment tools. Pathways were ranked by false discovery rate (FDR), and top terms were visualized to highlight processes associated with extracellular matrix remodeling, epithelial–mesenchymal transition, antigen processing and presentation, and interferon signaling.

### 2.9. Statistical Analysis

All statistical calculations were performed by R software, version 4.2.3. Adjusted *p* values < 0.05 were considered statistically significant for differential expression analysis. For comparisons among TNBC, HER2+, and ER+ within individual cell types (e.g., plasmablasts), we applied either t-tests or one-way ANOVA, as appropriate, followed by Tukey’s post hoc tests. All plots reported either adjusted *p*-values or formatted significance levels (e.g., *p* < 0.001). Plotting was performed using the *ggpubr*, *ggsignif*, and stat_compare_means functions from the *rstatix* package. Correlation heatmaps were generated with corrplot, reporting both correlation coefficients and significance levels.

### 2.10. Statistical Thresholds and Robustness Criteria

Differential expression analyses were performed using two-sided Wilcoxon rank-sum tests as implemented in Seurat’s FindMarkers function. *p*-values were adjusted for multiple testing using the Benjamini–Hochberg false discovery rate (FDR) correction. Genes were considered significantly differentially expressed if they met both of the following criteria: an absolute log_2_ fold-change ≥ 0.5 and an adjusted *p*-value (FDR) < 0.05, unless otherwise stated.

Within the bi-volcano framework, robustness was defined by directional concordance across independent subtype comparisons rather than effect size alone. Specifically, genes were prioritized as TNBC-enriched only if they exhibited positive log_2_ fold-change values (≥0.5) in both TNBC versus ER+ and TNBC versus HER2+ comparisons, with statistical significance maintained in at least one comparison and a consistent directional trend in the other. This dual-comparison requirement reduces sensitivity to noise, batch effects, and subtype-specific sampling variability by requiring reproducibility across biologically distinct reference groups.

For functional enrichment analyses, Gene Ontology and pathway enrichment were performed using the clusterProfiler package (v4.0.5). Enrichment analyses were conducted on significantly upregulated gene sets using the following parameters: ontology = “ALL”, *p*-value adjustment method = “BH”, *p*-value cutoff = 0.01, and q-value cutoff = 0.05.

## 3. Results

### 3.1. Identification of TNBC-Specific Gene Programs in Cancer Epithelial Cells

**Figure 1:**
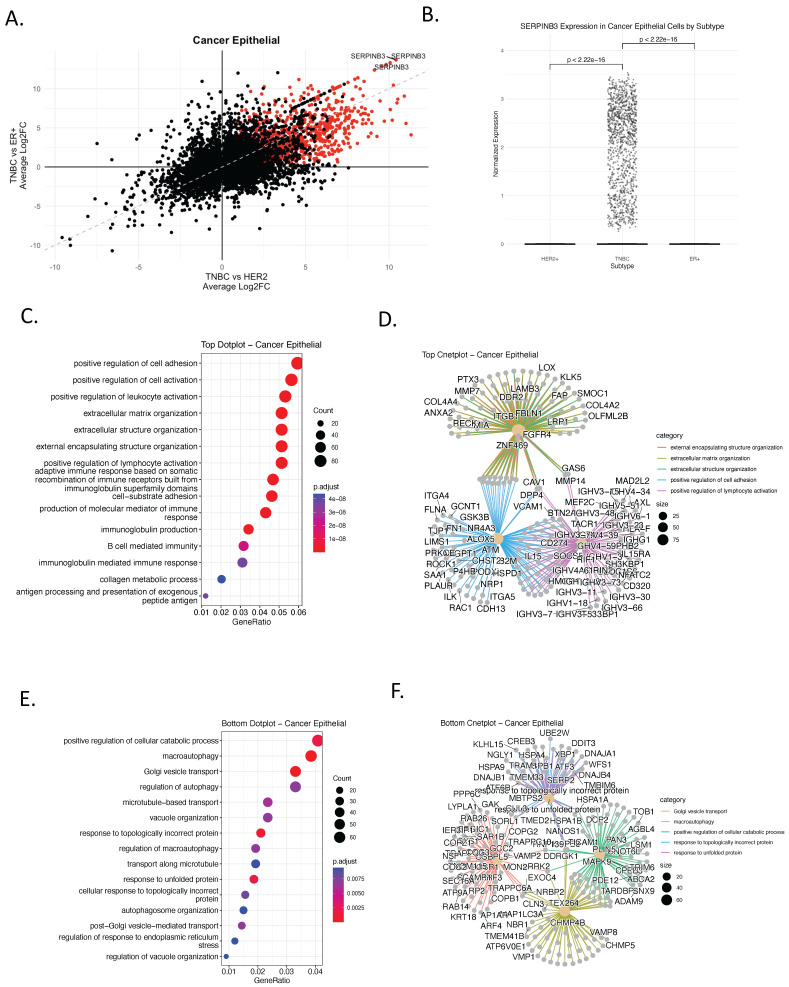
Identification of TNBC-Specific Gene Signatures and Pathway Enrichment in Cancer Epithelial Cells. (**A**) Bi-volcano plot comparing gene expression differences between TNBC and other breast cancer subtypes (HER2+ and ER+) in cancer epithelial cells, using single-cell RNA-sequencing (scRNA-seq) data from human primary breast cancer tumors [6]. Each dot represents a gene; those upregulated in TNBC (log_2_FC > 0.5, adjusted *p* < 0.05 in both comparisons) are colored red. The x-axis shows average log_2_ fold change (log_2_FC) between TNBC and ER+ tumors, while the y-axis shows log_2_FC between TNBC and HER2+ tumors. Genes in the upper right quadrant are significantly upregulated in TNBC compared to both HER2+ and ER+ tumors, highlighting TNBC-specific gene expression programs. In contrast, genes in the lower left quadrant are significantly downregulated in TNBC compared to both HER2+ and ER+, indicating genes specifically suppressed in TNBC. (**B**) Boxplot of SERPINB3 expression levels in cancer epithelial cells across subtypes. TNBC exhibits significantly higher expression compared to HER2+ and ER+ (*t*-test, *p* < 2.22 × 10^−16^) (**C**) Gene Ontology (GO) enrichment of TNBC-upregulated genes, showing biological processes. Color indicates adjusted *p*-value; dot size reflects gene count per term. (**D**) cnetplot of upregulated TNBC gene sets and their pathway associations. Only pathways with an adjusted *p*-value (FDR) < 0.05 were retained. (**E**) GO enrichment of TNBC-downregulated genes, highlighting suppressed processes including autophagy and exocytosis. (**F**) cnetplot of TNBC-downregulated genes showing network-level relationships among enriched pathways.

To identify gene programs unique to TNBC, we conducted differential gene expression analysis of the cancer epithelial subset using single-cell RNA-seq data from primary human breast tumors (*n* = 26) [9], comparing TNBC (*n* = 9) with HER2+ (*n* = 4) and ER+ (*n* = 13) subtypes. To enhance interpretability, we developed a bi-volcano plot framework—a novel visualization strategy that simultaneously projects fold-changes from two subtype comparisons onto orthogonal axes. In this representation, the x-axis denotes the log2 fold change for TNBC versus ER+, while the y-axis denotes the log2 fold change for TNBC versus HER2+ (Figure 1A, Supplementary Dataset Tables S1–S3). Unlike conventional volcano plots that assess one contrast at a time, this bi-dimensional design enables the direct identification of genes consistently enriched or depleted in TNBC relative to both receptor-positive subtypes. This plot strategy facilitates the interpretation of subtype-specific transcriptional profiles, in which genes located in the upper-right quadrant are upregulated in TNBC relative to both HER2+ and ER+, identifying them as TNBC-specific markers. Conversely, genes in the lower-left quadrant are downregulated in TNBC compared with both subtypes. This technique provides an intuitive and statistically robust method to highlight subtype-specific signatures, improving clarity in high-dimensional single-cell datasets.

This approach enabled the identification of genes whose overexpression is exclusive to TNBC, reinforcing their potential as subtype-specific biomarkers and therapeutic targets. The analysis revealed a distinct cluster of genes selectively upregulated in TNBC. Among these, SERPINB3 emerged as a prominent candidate, exhibiting specific and significant overexpression in TNBC epithelial cells compared to HER2+ and ER+ tumors (Figure 1A,B and Supplementary dataset). *SERPINB3* encodes a serine protease inhibitor implicated in modulating apoptosis, inflammation, and immune evasion in various cancers [15], making it a compelling candidate for targeted therapy in TNBC. In the context of this analysis, SERPINB3 was identified as a TNBC-enriched epithelial biomarker candidate, rather than a universal TNBC feature, motivating further validation.

To understand the biological context of the TNBC-specific transcriptional programs, we conducted Gene Ontology (GO) enrichment analysis on both the upregulated (Figure 1C,D) and downregulated (Figure 1E,F) gene sets identified from the bi-volcano comparison. The genes upregulated in TNBC were associated with biological processes such as cell adhesion, leukocyte activation, extracellular matrix organization, and notably, B-cell-mediated immunity and immunoglobulin production (Figure 1C). Network visualization highlighted key gene hubs involved in these processes, including *FBLN1*, *CAV1*, *FGFR4*, and *ITGB1*, suggesting a possible role for TNBC epithelial cells in modulating the tumor immune microenvironment (Figure 1D). In contrast, genes downregulated in TNBC were enriched for autophagy, vesicle organization, and protein folding stress responses (Figure 1E), indicating suppression of homeostatic and metabolic pathways. Functional network analysis further identified *ATM*, *HSPD1*, *ARPC2*, and *ILK* as core contributors to these downregulated processes (Figure 1F).

To further characterize the transcriptional phenotype of TNBC, we performed Gene Set Enrichment Analysis (GSEA) using Hallmark gene sets representing key oncogenic pathways (Supplementary Figure S1). TNBC epithelial cells showed significant enrichment for epithelial–mesenchymal transition (EMT), inflammatory signaling, interferon responses, and MYC target pathways. In contrast, ER+ and HER2+ tumors exhibited enrichment for estrogen response pathways, consistent with their hormone-driven biology (Supplementary Figure S1). Collectively, these results define a TNBC-specific epithelial transcriptional landscape characterized by immune-associated and mesenchymal-like programs, providing a foundation for downstream validation and biomarker-oriented analyses.

### 3.2. Independent Clinical Validation of SERPINB3 and TNBC-Specific Pathway Programs Using the METABRIC Cohort

**Figure 2:**
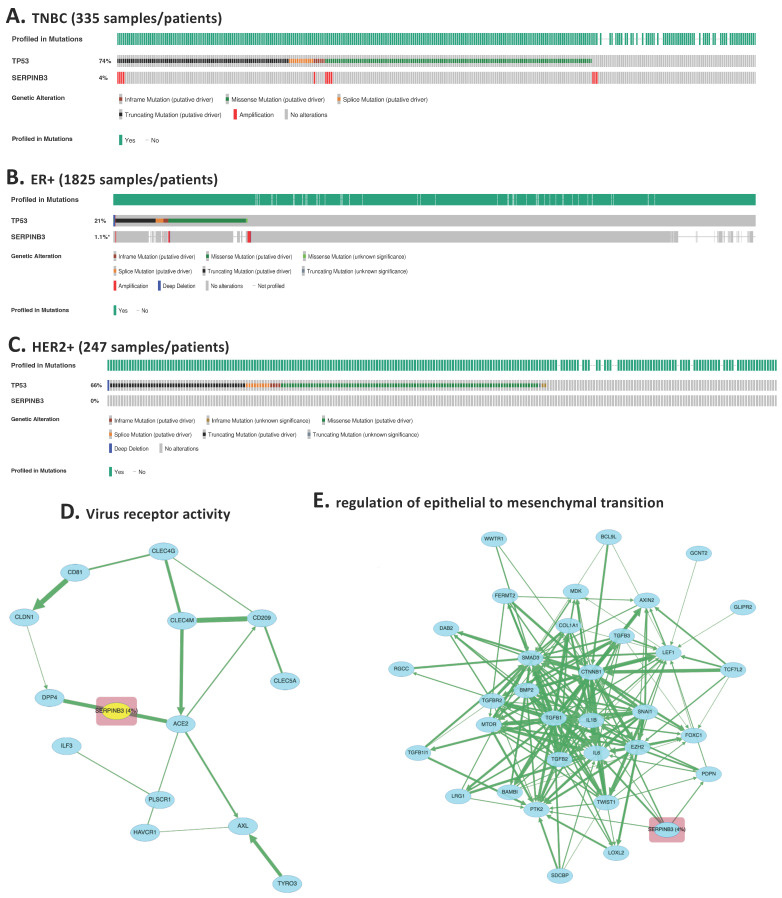
Independent clinical validation of SERPINB3 overexpression and TNBC-specific pathway activation using the METABRIC cohort. (**A**–**C**) SERPINB3 expression across >2400 primary breast cancers in the METABRIC dataset, stratified by molecular subtype. Heatmap-style tracks demonstrate that SERPINB3 is overexpressed almost exclusively in TNBC tumors (4% of 335 TNBC cases), with minimal expression in ER+ tumors (1.1% of 1825 cases) and no detectable expression in HER2+ tumors (0% of 247 cases) *: *p* < 0.05. (**D**) Pathway analysis of SERPINB3 was performed using the cBioPortal “Enrichment” and “Pathway Analysis” modules, restricted to TNBC samples and ranked by *p*-value significance. The top enriched pathways, “virus receptor activity”, emerged prominently. (**E**) The second top enriched pathways enriched in SERPINB3-altered TNBC tumors is epithelial–mesenchymal transition (EMT). The clustering of SERPINB3 within these biologically coherent modules supports its mechanistic role in TNBC epithelial remodeling and immune escape.

To assess whether the TNBC-enriched epithelial signals identified at single-cell resolution generalize to an independent clinical cohort, we examined SERPINB3 expression in the METABRIC dataset comprising 2509 primary breast tumors [10]. Consistent with the single-cell findings (Figure 1A,B), *SERPINB3* amplification demonstrated a TNBC-enriched pattern, with detectable amplification in 4% of TNBC cases (*n* = 335) compared with only 1% of ER+ tumors (*n* = 1825) and 0% of HER2+ tumors (*n* = 247) (Figure 2A–C). Although the absolute frequency within TNBC is modest, the complete absence or near-absence of expression in receptor-positive subtypes supports specificity rather than prevalence, consistent with a context-dependent epithelial biomarker.

To evaluate whether *SERPINB3*-expressing TNBC tumors exhibit pathway-level features concordant with the single-cell analysis, we performed pathway enrichment and interaction analyses using cBioPortal, restricted to TNBC cases. Among the top enriched categories, “virus receptor activity” emerged as a significantly associated pathway (Figure 2D), consistent with the immune-modulatory and stress-response processes identified in our GO enrichment analysis of TNBC-upregulated genes. This pathway involves genes linked to epithelial plasticity, cellular stress signaling, and innate immune sensing, supporting the multi-functional role of *SERPINB3* inferred from the single-cell dataset.

Expanded protein–protein interaction mapping revealed that SERPINB3 is embedded within interconnected modules related to EMT (Figure 2E). These pathway clusters align closely with the GSEA (Supplementary Figure S1), further confirming that SERPINB3 participates in transcriptional programs associated with mesenchymal remodeling, immune modulation, and TNBC aggressiveness.

Together, these analyses demonstrate that *SERPINB3* is reproducibly enriched in a subset of TNBC tumors across both single-cell and large-scale clinical datasets and is associated with pathway programs linked to epithelial plasticity and immune modulation. Such low-frequency but subtype-restricted expression patterns are increasingly recognized as clinically relevant in heterogeneous cancers, where molecularly defined subgroups may exhibit distinct biology and therapeutic vulnerabilities. These findings support SERPINB3 as a TNBC-enriched epithelial marker whose associated transcriptional programs warrant further functional and translational investigation, rather than as a definitive driver of TNBC biology.

### 3.3. TNBC-Specific Activation of Immunoglobulin and B Cell Pathways in Plasmablasts and B Cells

**Figure 3:**
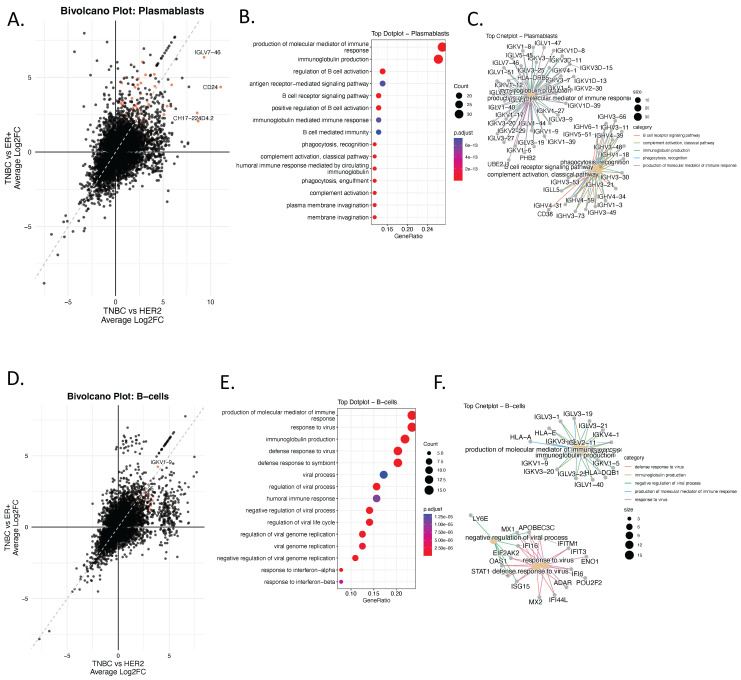
Ectopic Expression of Immunoglobulin Genes in Non-B Cells Reveals TNBC-Specific Immune Mimicry Signatures. (**A**) Bi-volcano plot showing differentially expressed genes (DEGs) in plasmablasts comparing TNBC to ER+ (x-axis) and TNBC to HER2+ (y-axis). Genes upregulated in TNBC compared to both subtypes are highlighted in red. (**B**) Dot plot displaying enriched biological processes (GO:BP) associated with the top TNBC-upregulated genes in plasmablasts. Color indicates adjusted *p*-value; dot size reflects gene count per term. (**C**) Corresponding cnetplot visualizing the top enriched pathways and their associated genes in plasmablasts. Only pathways with an adjusted *p*-value (FDR) < 0.05 were retained. (**D**), Bi-volcano plot showing differentially expressed genes (DEGs) in B-Cells comparing TNBC to ER+ (x-axis) and TNBC to HER2+ (y-axis). Genes upregulated in TNBC compared to both subtypes are highlighted in red. (**E**) GO enrichment of upregulated DEGs, and (**F**) cnetplot showing enriched immunoglobulin-related immune processes.

To investigate immune remodeling associated with TNBC, we performed differential gene expression and enrichment analyses focused specifically on immune cell populations, including plasmablasts and B cells using single-cell RNA-seq data. Bi-volcano plots revealed a distinct cluster of genes upregulated exclusively in TNBC, including immunoglobulin genes such as *IGLV3-25*, *IGKV1-9*, and *IGLV1-40*, which were not upregulated in HER2+ or ER+ tumors (Figure 3A–F and Supplementary dataset Tables S1 and S2). These genes are typically restricted to antibody-producing B lineage cells, suggesting aberrant or heightened B cell and plasmablast activity in TNBC.

Functional enrichment analysis of genes upregulated in TNBC plasmablasts showed strong enrichment for humoral immune responses (Figure 3B), including immunoglobulin production, B cell receptor signaling, complement activation, and antigen receptor–mediated signaling. These results were further supported by category netplot (cnetplots) demonstrating high connectivity between immunoglobulin genes and humoral or adaptive immune–related GO terms (Figure 3C).

Similarly, TNBC B cells exhibited overrepresentation of genes involved in viral response, immunoglobulin production, and interferon signaling (Figure 3E,F). These enrichments were largely absent in HER2+ and ER+ tumors, supporting the existence of a unique B-cell-driven immune axis in TNBC.

Collectively, these findings indicate a TNBC-specific activation of adaptive immune and antibody-mediated programs within B cells and plasmablasts. This reflects an immune-activated tumor microenvironment or, alternatively, the emergence of tumor-associated B-like transcriptional signatures.

### 3.4. Ectopic Expression of Immunoglobulin Genes in Non-B Cells Reveals TNBC-Specific Immune Mimicry Signatures

**Figure 4:**
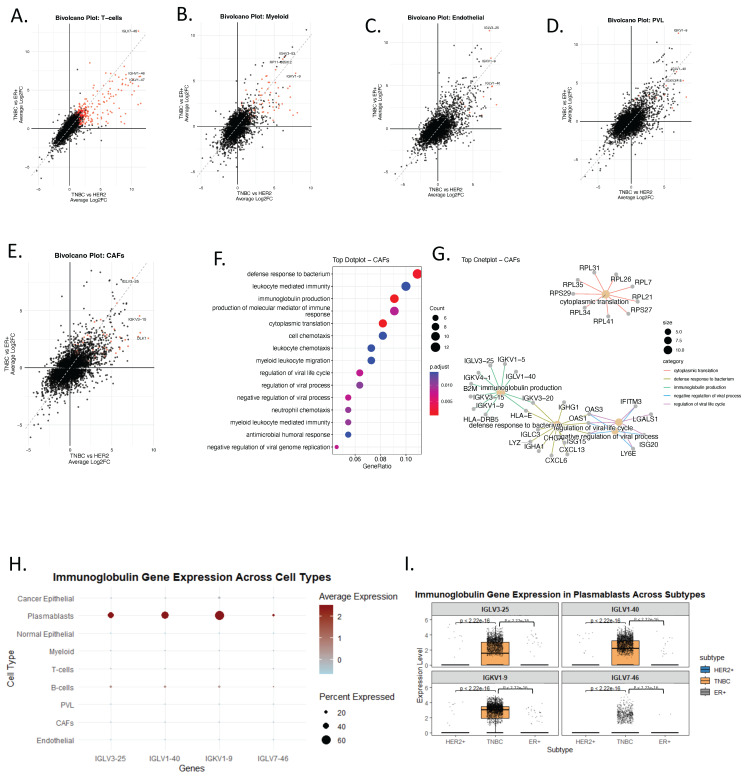
Ectopic Expression of Immunoglobulin Genes in Non-B Cells Reveals TNBC-Specific Immune Mimicry Signatures. (**A**–**E**) Bi-volcano plots for TNBC vs. HER2+ (x-axis) and TNBC vs. ER+ (y-axis) comparisons across five cell populations: T-cells (**A**), myeloid (**B**), endothelial (**C**), PVL (**D**), and CAFs (**E**). Red dots mark genes significantly upregulated in TNBC in both comparisons. (**F**) Dot plot displaying GO enrichment analysis associated with the top TNBC-upregulated genes in CAFs. Color indicates adjusted *p*-value; dot size reflects gene count per term. (**G**) cnetplot of upregulated TNBC gene sets in CAFs and their pathway associations. Only pathways with an adjusted *p*-value (FDR) < 0.05 were retained. (**H**) Dot plot showing pseudo-bulk average expression (color), and percent of cells expressing (dot size) immunoglobulin genes across all major cell types. (**I**) Boxplots comparing immunoglobulin gene expression in plasmablasts across subtypes (HER2+, TNBC, ER+), showing significantly higher expression of IGLV3-25, IGLV1-40, and IGKV1-9 in TNBC.

To further examine tumor microenvironment–specific transcriptional reprogramming in TNBC, we performed bi-volcano analyses comparing TNBC with HER2+ and ER+ subtypes across several major cell lineages, including T cells, cancer-associated fibroblasts (CAFs), myeloid cells, perivascular-like (PVL) cells, and endothelial cells. Notably, the analysis revealed that the most prominent TNBC-specific programs involved ectopic immunoglobulin gene expression, extending beyond classical B-lineage compartments (Figure 4A–G, Supplementary Figure S2 and Supplementary dataset Tables S1 and S2).

Among the top upregulated genes, *IGLV3-25*, *IGLV1-40*, *IGKV1-9*, and *IGLV7-46* were consistently enriched across multiple non-B cell types in TNBC (Figure 4A–G). To confirm that immunoglobulin gene expression is not a typical feature of non-B cells, we generated a dot plot summarizing pseudo-bulk gene expression across cell types and molecular subtypes (Figure 4G). As expected, immunoglobulin genes were highly and specifically expressed in plasmablasts (Figure 4G), whereas detectable expression of these genes in non-B cells was observed only in TNBC (Figure 4A–G). Subtype-specific analysis of the plasmablast compartment further confirmed significantly elevated expression of immunoglobulin genes in TNBC only, with highly significant *p*-values relative to HER2+ and ER+ tumors (Figure 4H).

Several potential technical confounders were considered. First, ambient RNA contamination would be expected to produce diffuse; however, the observed expression was subtype-restricted and selectively enriched in TNBC. Second, technical doublets or cell-type misclassification would likely result in inconsistent or stochastic patterns, whereas the immunoglobulin signature was reproducibly detected across multiple stromal and endothelial populations of only TNBC subtype. Third, stress-response–associated transcriptional artifacts typically induce heat-shock or immediate-early gene programs rather than coordinated enrichment of immunoglobulin variable region genes. Together, these observations make technical leakage or stress artifacts an unlikely explanation for the observed patterns.

These findings support the existence of immune mimicry in TNBC, in which non-B cells aberrantly express immunoglobulin-related genes. This ectopic expression may contribute to immune evasion and suppression.

CAF-specific GO enrichment analysis (Figure 4F,G) revealed upregulation of leukocyte chemotaxis, antimicrobial humoral responses, and immunoglobulin-mediated immunity, reinforcing the notion that stromal cells in TNBC may adopt immune-like functions. Similarly, endothelial cells in TNBC showed enrichment in immunoglobulin production and antigen processing pathways (Supplementary Figure S2C), further supporting the idea that non-B cell compartments may participate in immune mimicry. These findings identify a TNBC-specific immune-mimicry signature in non-B cell compartments that may serve as a biomarker of immune dysregulation within the tumor microenvironment.

Ectopic immunoglobulin gene expression in non-lymphoid cells highlights a reproducible transcriptional state associated with TNBC aggressiveness and immune remodeling. While the functional consequences remain to be determined, this immune-mimicry signature provides a testable hypothesis linking stromal reprogramming to immune evasion and warrants further mechanistic and translational investigation.

### 3.5. Imaging-Based Validation of Immune Suppression and Stromal Reprogramming in TNBC

To validate, at the tissue level, the TNBC-specific tumor microenvironment (TME) reprogramming identified by scRNA-seq, we analyzed a multiplexed imaging cohort of TNBC tumors (*n* = 38, 49 markers) [11]. This analysis was designed to provide orthogonal, protein-level evidence for coordinated immune and stromal states inferred from transcriptional data, rather than to establish direct causal mechanisms. Composite indices were generated to quantify immune suppression (PD-L1, PD-1, LAG3, FOXP3, IDO, CD68), CAF activation (SMA, Vimentin), endothelial activation (CD31), and tumor proliferation (Ki67). These indices enabled integrated assessment of immune, stromal, and epithelial states within the TNBC TME.

**Figure 5:**
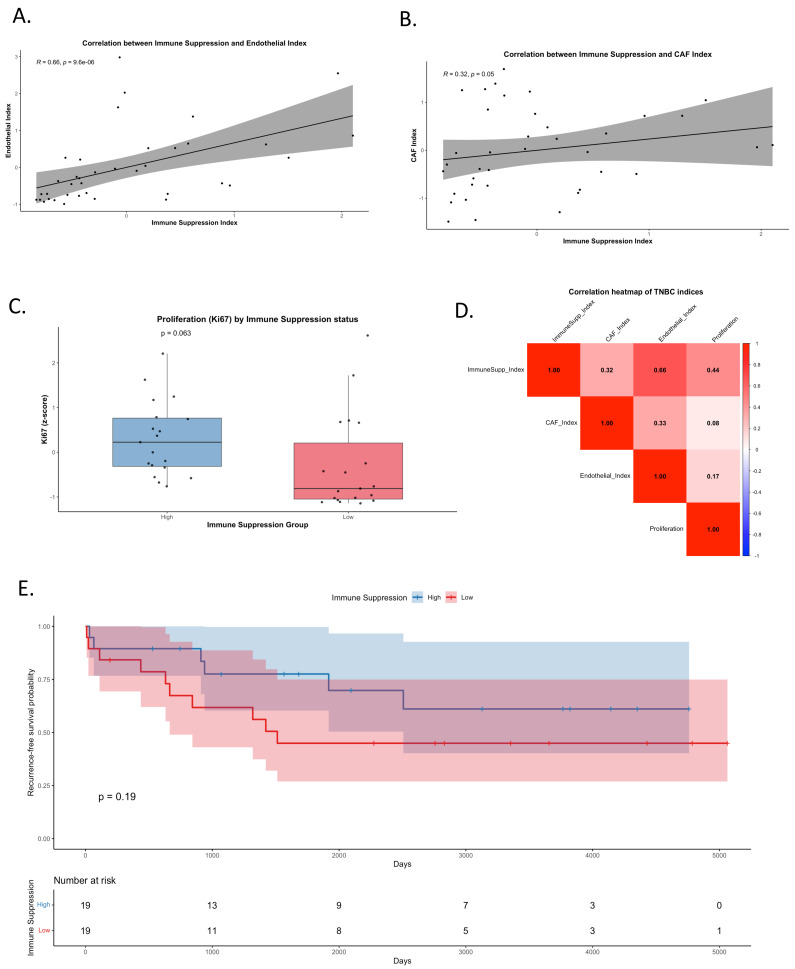
Crosstalk between immune suppression, stromal reprogramming, and tumor progression in TNBC. (**A**) Correlation between the Immune Suppression Index (PD-L1, PD-1, LAG3, FOXP3, IDO, CD68) and Endothelial Index (CD31). A strong positive association was observed (Spearman R = 0.66, *p* < 0.001). (**B**) Correlation between the Immune Suppression Index and CAF Index (SMA, Vimentin), showing a weaker but notable positive correlation (R = 0.32, *p* = 0.05). (**C**) Boxplot of Ki67 (proliferation marker) z-scores stratified by high versus low immune suppression groups, showing a trend toward higher proliferation in immune-suppressed tumors (*p* = 0.063). (**D**) Heatmap of pairwise correlations among Immune Suppression, CAF, Endothelial, and Proliferation indices. Strongest correlations were observed between immune suppression and endothelial activity, and between immune suppression and proliferation. (**E**) Kaplan–Meier curve of recurrence-free survival (RFS) stratified by immune suppression status (high vs. low, median split).

Strong positive correlations were observed between the Immune Suppression Index and endothelial activation (Spearman R = 0.66, *p* < 0.001; Figure 5A), indicating that TNBC tumors with heightened immune-suppressive signaling also exhibit pronounced endothelial remodeling. A weaker but notable correlation with CAF activation (R = 0.32, *p* = 0.05; Figure 5B) further supports the concept of coordinated stromal reprogramming contributing to immune-evasive niches in TNBC. Importantly, these associations reflect co-occurring phenotypic states rather than direct stromal–immune signaling dependencies, and can be interpreted as spatially resolved correlates of immune suppression within the TNBC TME.

Functionally, tumors with high immune suppression demonstrated elevated proliferation, as reflected by increased Ki67 levels (*p* = 0.063; Figure 5C), consistent with the hypothesis that immune-suppressive microenvironments may permit increased tumor growth. A global correlation heatmap confirmed these associations, showing the strongest relationships between immune suppression, endothelial activation, and proliferation (Figure 5D and Supplementary dataset Table S4). Together, these patterns identify a reproducible imaging-based signature linking immune suppression with stromal activation and proliferative state in TNBC.

Ultimately, Kaplan–Meier analysis demonstrated that while tumors with high immune suppression trended toward higher recurrence-free survival (RFS), the difference did not reach statistical significance (*p* = 0.19; Figure 5E). These findings underscore that immune suppression alone does not significantly stratify survival outcomes, but its association with stromal reprogramming and proliferation supports its role in shaping the aggressive TNBC microenvironment. These imaging-based findings serve to validate the presence of coordinated immune–stromal phenotypes at the tissue level that parallel transcriptional programs identified by scRNA-seq.

The multiplex imaging analysis provides independent, spatially resolved support for TNBC-associated immune suppression and stromal reprogramming. While causal mechanisms cannot be inferred from correlation analyses alone, the convergence of transcriptional and imaging data strengthens the evidence for a coupled immune–stromal state that may contribute to TNBC aggressiveness and warrants further mechanistic and translational investigation.

### 3.6. SERPINB3-Centered Protein–Protein Interaction Analysis Reveals Mechanistic Links Between Tumor-Intrinsic Programs and Stromal Immune Suppression

After validating the single-cell findings at the tumor-intrinsic level using cBioPortal clinical data, and confirming cell-extrinsic stromal remodeling using multiplex imaging, we next sought to determine whether SERPINB3 itself could mechanistically explain the coordinated stromal activation and immune suppression observed in TNBC. To address this, we input the TNBC-exclusive cancer epithelial gene set identified from the scRNA-seq analysis (Supplementary dataset Table S3) into the STRING protein–protein interaction database and generated a SERPINB3-centered interaction network (Supplementary dataset Table S5).

**Figure 6:**
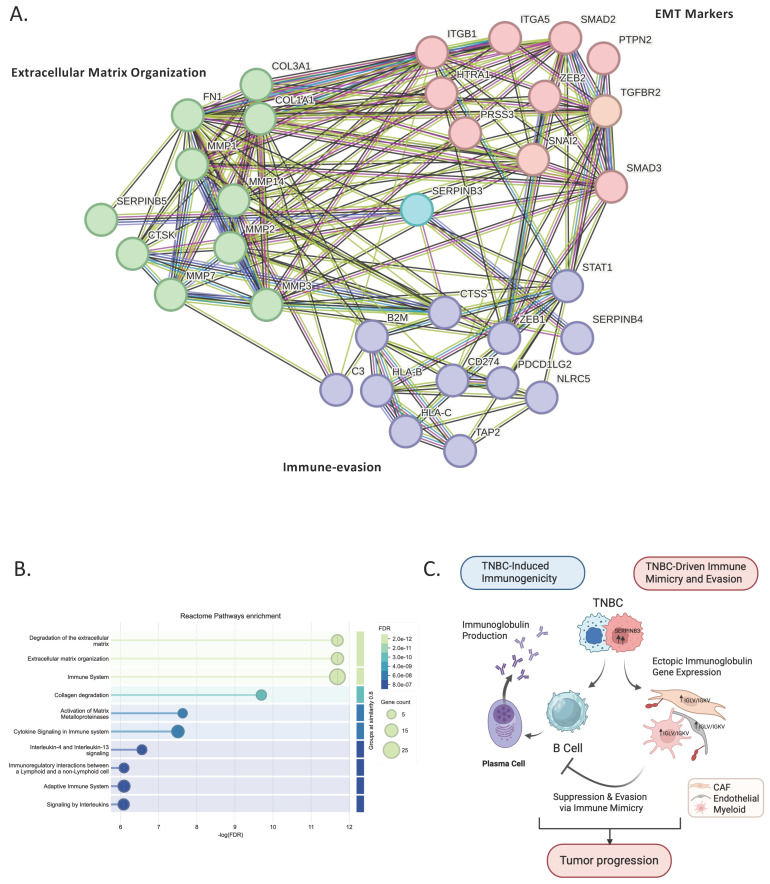
SERPINB-centered protein–protein interaction network and pathway enrichment of TNBC-exclusive cancer epithelial genes. (**A**) STRING protein–protein interaction network generated from TNBC-exclusive epithelial genes identified in the single-cell analysis (Supplementary dataset). SERPINB3 emerges as a central hub linking clusters involved in extracellular matrix remodeling (COL1A1/2, FN1, MMPs), EMT regulation (ZEB1/2, SNAI2, SMADs), and immune-modulatory pathways (STAT1, NLRC5, HLA class I genes). (**B**) GO Biological Process enrichment of the SERPINB-linked network showing the top significant pathways, including ECM organization, EMT, antigen processing and presentation, and interferon-γ–related immune signaling. Dot size reflects gene count and color indicates FDR. (**C**) Schematic model illustrating the dual role of TNBC in shaping tumor immunity. TNBC enhances B-cell and plasmablast activation, leading to increased immunoglobulin production and heightened immunogenicity. Conversely, TNBC epithelial cells upregulate SERPINB3, driving stromal reprogramming toward immune suppression; CAF, endothelial, and myeloid cells acquire immune-mimicry features through ectopic IGLV/IGKV expression. This stromal mimicry suppresses authentic B-cell activity and promotes immune evasion, collectively supporting tumor progression. Figure created with BioRender.com.

The resulting network (Figure 6A) revealed that SERPINB3 occupies a highly connected position linking multiple biological modules relevant to TNBC pathobiology. SERPINB3 was directly associated with clusters involved in extracellular matrix remodeling (MMP family, COL1A1/2, FN1), epithelial–mesenchymal transition (ZEB1/2, SNAI2, SMAD2/3/4), and immune-evasion pathways including interferon signaling, antigen presentation (HLA class I genes, B2M, TAP2), and innate immune regulation (STAT1, NLRC5). These interconnected modules mirror the biological processes identified in the GO and pathway enrichment results from the single-cell analysis, further supporting SERPINB3 as a potential driver of TNBC-specific transcriptional remodeling.

Pathway enrichment of the SERPINB-linked cluster (Figure 6B) demonstrated significant overrepresentation of mesenchymal transition, extracellular matrix organization, and interferon-γ–mediated immune regulation. Together, these pathways recapitulate the stromal and immune features validated in the imaging dataset, indicating that SERPINB3 may function as a molecular bridge connecting tumor-intrinsic programs with stromal reprogramming and immune suppression.

Finally, we integrated these findings into a working schematic model (Figure 6C), proposing how TNBC may orchestrate a dual immune phenotype. On one side, TNBC is associated with B-cell and plasmablast activation, resulting in increased immunoglobulin production and a more immunogenic tumor context. Conversely, TNBC epithelial cells exhibit upregulation of SERPINB3 and are associated with stromal reprogramming toward immune suppression, in which CAFs, endothelial cells, and myeloid populations display immune-mimicry features through ectopic IGLV/IGKV gene expression. Rather than establishing a definitive mechanism, this model suggests that stromal immune mimicry may attenuate authentic B-cell activity and contribute to immune evasion. Taken together, the data support a conceptual framework in which TNBC tumors may simultaneously engage immune activation and immune suppression, offering a hypothesis to explain their paradoxical immunogenic yet aggressive behavior.

## 4. Discussion

This study integrates single-cell transcriptomics, large-scale clinical data, and multiplexed tissue imaging to identify and validate tumor-intrinsic and tumor-extrinsic mechanisms that collectively shape the complex immune landscape of triple-negative breast cancer (TNBC). By combining these complementary data sources, we delineate a dual program in which TNBC simultaneously engages immune activation and immune evasion pathways, ultimately fostering an aggressive and therapeutically challenging tumor microenvironment (TME).

Single-cell analysis revealed that TNBC epithelial cells harbor a distinct transcriptional program characterized by strong enrichment of inflammatory signaling, interferon responses, epithelial–mesenchymal transition (EMT), and extracellular matrix remodeling. Among the most prominent TNBC-specific genes, SERPINB3 emerged as a consistent and highly upregulated epithelial marker, with minimal or absent expression in HER2+ and ER+ tumors. SERPINB3 (SCCA1) has been implicated in promoting tumor progression, EMT, and immune evasion in hepatocellular carcinoma, cervical cancer, and lung cancer [16,17,18]. Mechanistically, SERPINB3 inhibits apoptosis, modulates cytokine secretion, and contributes to hypoxic niche formation [19]; all features aligned with the aggressive phenotype observed in TNBC. To independently validate tumor-intrinsic findings, we analyzed the METABRIC dataset of 2509 primary breast tumors. SERPINB3 expression was detected in 4% of TNBC cases but was nearly absent in ER+ and HER2+ tumors (1% and 0%, respectively). Pathway enrichment of SERPINB3-altered TNBC cases further confirmed activation of EMT and immune-evasion programs, reinforcing SERPINB3 as a clinically relevant TNBC-specific epithelial driver. This independent validation supports the robustness and translational relevance of the scRNA-seq findings.

In addition to tumor-intrinsic programs, scRNA-seq revealed a striking TNBC-specific expansion of plasmablasts and B cells, accompanied by elevated immunoglobulin gene expression. These findings are consistent with reports linking B-cell–driven immunity and tertiary lymphoid structures to enhanced immunotherapy responsiveness [20,21]. Unexpectedly, immunoglobulin genes were also ectopically expressed in non-B cell populations—including CAFs, endothelial cells, and myeloid cells—exclusively in TNBC. This phenomenon reflects immune mimicry, an emerging mechanism by which stromal or tumor cells co-opt immune gene programs to evade detection or modulate local immunity [22,23]. Prior evidence suggests that such mimicry may contribute to resistance to immune checkpoint blockade [24,25,26], positioning this finding as a potentially clinically meaningful feature of TNBC biology.

Multiplexed imaging provided tissue-level validation for the scRNA-seq–derived tumor-extrinsic programs. Strong correlations between immune suppression (PD-L1, PD-1, LAG3, FOXP3, IDO, and CD68) and stromal activation (CAF and endothelial indices) indicate that TNBCs with immune-suppressive features also exhibit coordinated stromal remodeling. Tumors with high immune suppression also displayed increased proliferation, aligning with the aggressive phenotype predicted by single-cell data. These observations mirror prior studies demonstrating that CAF heterogeneity and stromal–immune spatial patterns strongly influence immune cell infiltration and clinical outcomes in TNBC [27,28,29,30,31].

To mechanistically connect tumor-intrinsic SERPINB3 activity with the stromal immune suppression observed in TNBC, TNBC-exclusive epithelial genes were analyzed using STRING. The resulting SERPINB3-centered network revealed dense interactions with ECM remodeling genes (MMPs, COL1A1/2, FN1), EMT regulators (ZEB1/2, SNAI2, SMAD2/3/4), and immune-evasion pathways including interferon signaling and antigen presentation (HLA class I, B2M, TAP2). Pathway enrichment confirmed overrepresentation of EMT, ECM organization, antigen-processing pathways, and interferon-γ responses, mirroring both the scRNA-seq and imaging findings. These results suggest that SERPINB3 may function as a molecular bridge that links tumor-intrinsic signaling programs to stromal activation and immune evasion.

Bringing together these multi-platform analyses, we propose a working model in which TNBC may exhibit a dual immunological phenotype (Figure 6C). On one side, TNBC promotes B-cell and plasmablast activation, generating heightened immunogenicity. On the other hand, SERPINB3 upregulation in TNBC epithelial cells is associated with stromal remodeling, including CAF, endothelial, and myeloid immune-mimicry signatures characterized by ectopic immunoglobulin gene expression. Rather than establishing direct causality, this immune mimicry is hypothesized to attenuate authentic B-cell function and contribute to immune evasion. Together, these observations support a conceptual framework in which immune stimulation and immune suppression coexist within the TNBC tumor microenvironment, potentially shaping an ecosystem permissive to tumor progression despite active immune engagement.

Importantly, the dual-phenotype framework proposed in this study should be interpreted as a working model rather than a definitive mechanistic conclusion. While our integrative analyses identify robust associations between epithelial SERPINB3 expression, stromal immune mimicry, and immune-suppressive tumor microenvironments, functional experiments will be required to establish causality. Specifically, future studies are needed to determine whether SERPINB3 directly regulates stromal immune behavior, whether ectopic immunoglobulin expression in non-lymphoid compartments actively alters immune signaling, and whether these features predict therapeutic response or resistance. The absence of perturbation-based validation (e.g., genetic or pharmacologic modulation) represents an important limitation of the current study. From a translational perspective, the identified signatures—including SERPINB3 enrichment and non-lymphoid immunoglobulin expression—should therefore be viewed as candidate biomarkers rather than confirmed therapeutic targets. We anticipate that integrative biomarker frameworks capturing both immune engagement and immune evasion, rather than immune activation alone, will be essential for optimizing patient stratification and combination immunotherapy strategies in TNBC.

## 5. Conclusions

This study advances the understanding of TNBC biology by leveraging multi-modal single-cell, clinical, and spatial analyses to identify TNBC-specific molecular programs that link epithelial transcriptional reprogramming to immune suppression and stromal immune mimicry, thereby advancing immune-target discovery beyond descriptive immune profiling. Our analyses address a long-standing question in the field—how TNBC can be simultaneously immune-active yet clinically aggressive—by demonstrating the coexistence of immune activation and immune-suppressive features rather than a single dominant immune state. At the data-supported level, we demonstrate enrichment of humoral immune programs and TNBC-restricted SERPINB3 expression, alongside coordinated stromal and immune-suppressive phenotypes at the tissue level. At the inferential level, we propose that the convergence of epithelial SERPINB3-associated programs and stromal immune mimicry may represent one mechanism through which immune activation fails to translate into durable tumor control. Together, these findings provide a framework for future biomarker development and rational combination immunotherapy strategies in TNBC.

## Data Availability

The scRNA-seq publicly available data used in this study are available through the Gene Expression Omnibus under accession number GSE176078 [6]. The METABRIC cohort, a transcriptomic and clinical dataset of 2509 primary breast cancers (TNBC = 335, ER+ = 1825, HER2+ = 247) was accessed via cBioPortal (https://www.cbioportal.org/study/summary?id=brca_metabric, accessed on 20 Noveber 2025) [10]. A multiplexed tissue imaging publicly available data of TNBC tumors used in this study are available at [8]. All analyses were performed using R version 4.2.3 with established, publicly available R packages. All data generated in this study are available in the Supplementary dataset Tables S1–S5.

## Supplementary Materials

The following supporting information can be downloaded at https://www.mdpi.com/article/10.3390/genes17010038/s1, Figure S1: Hallmark gene set enrichment analysis (GSEA) of TNBC versus other breast cancer subtypes; Figure S2: Gene Ontology Enrichment of TNBC-Upregulated Genes Across Immune and Stromal Cell Types. Supplementary dataset: Table S1: TNBC_vs._ER_markers; Table S2: TNBC_vs._HER2_markers; Table S3: TNBC_upregulated_genes_in_cancer; Table S4: Correlation_Pvalues_TNBC; Table S5: SERPINB3-centered interaction network.

## Abbreviations

The following abbreviations are used in this manuscript:

TNBC	Triple-Negative Breast Cancer
ER+	Estrogen Receptor–Positive
HER2+	Human Epidermal Growth Factor Receptor 2–Positive
scRNA-seq	Single-Cell RNA Sequencing
