# Supplementary material for: Multi-Modal Profiling Reveals SERPINB3-Driven Immune Evasion and Stromal Immune Mimicry in Triple-Negative Breast Cancer
Source: Genes (Basel). 2025 Dec 31;17(1):38. doi: 10.3390/genes17010038 (PMC12840653; doi:10.3390/genes17010038)
Supplement: Supplementary file 1 [file genes-17-00038-s001.zip › Supplementary Figure 1.pdf]

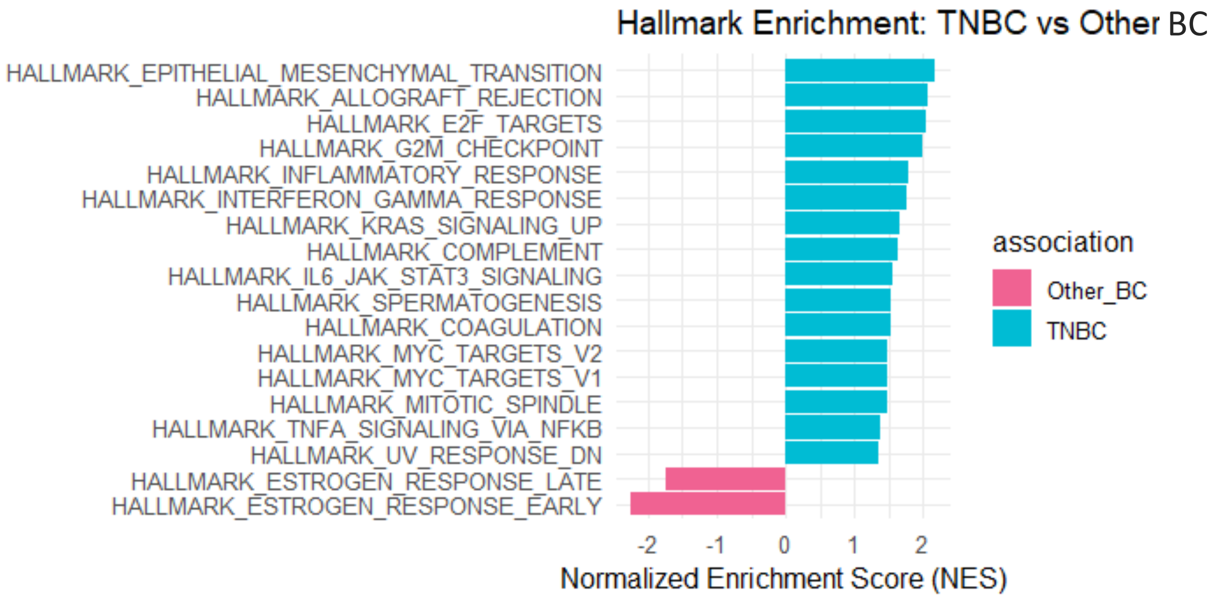

Supplementary Figure 1: Hallmark gene set enrichment analysis (GSEA) of TNBC versus other breast cancer subtypes. *Hallmark GSEA demonstrates that TNBC exhibits significant enrichment of immune-related pathways (inflammatory response, interferon-γ response, TNFα/NFκB signaling, complement activation, IL6/JAK/STAT3 signaling) and cell-cycle/mesenchymal transition programs (epithelial–mesenchymal transition, E2F targets, G2M checkpoint, MYC signaling). In contrast, hormone response pathways (estrogen response early/late) were selectively enriched in HER2+/ER+ tumors. Pathways with an adjusted p-value (FDR) < 0.05 were considered significant.*
