# Supplementary material for: Multi-Modal Profiling Reveals SERPINB3-Driven Immune Evasion and Stromal Immune Mimicry in Triple-Negative Breast Cancer
Source: Genes (Basel). 2025 Dec 31;17(1):38. doi: 10.3390/genes17010038 (PMC12840653; doi:10.3390/genes17010038)
Supplement: Supplementary file 1 [file genes-17-00038-s001.zip › Supplementary Figure 2.pdf]

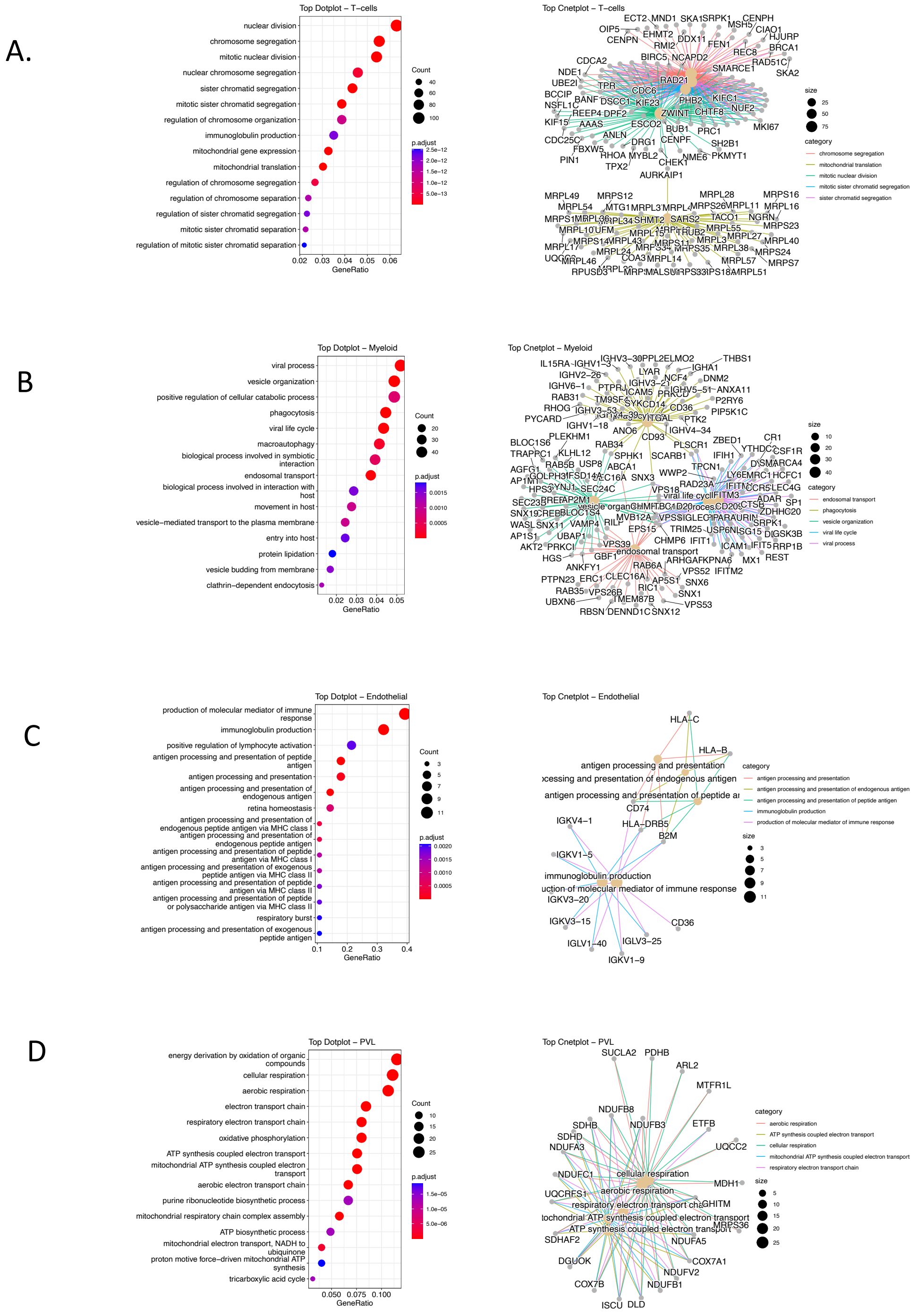

Supplementary Figure 2: Gene Ontology Enrichment of TNBC-Upregulated Genes Across Immune and Stromal Cell Types. Dotplots (left) and cnetplots (right) represent the top enriched GO Biological Process terms for genes upregulated in TNBC compared to HER2+ and ER+ tumors within each indicated cell type. **(A)** T cells, **(B)** Myeloid cells, **(C)** Endothelial cells, and **(D)** Perivascular-like (PVL) cells. GO terms were enriched using the clusterProfiler package, and significance was determined using Benjamini–Hochberg adjusted p-values. Dot size indicates gene count per term; color represents adjusted p-value significance. Cnetplots visualize gene–pathway connections, with color-coded categories reflecting pathway identity.
